# Generalization of fear of movement-related pain and avoidance behavior as predictors of work resumption after back surgery: a study protocol for a prospective study (WABS)

**DOI:** 10.1186/s40359-022-00736-5

**Published:** 2022-02-22

**Authors:** Rini Masuy, Lotte Bamelis, Katleen Bogaerts, Bart Depreitere, Kris De Smedt, Jeroen Ceuppens, Bert Lenaert, Sarah Lonneville, Dieter Peuskens, Johan Van Lerbeirghe, Patrick Van Schaeybroeck, Peter Vorlat, Steefka Zijlstra, Ann Meulders, Johan W. S. Vlaeyen

**Affiliations:** 1 Research Group Health Psychology, KU Leuven, Leuven, Belgium; 2 Centre for Translational Psychological Research TRACE, Genk, Belgium; 3 Department of Psychology, Ziekenhuis Oost-Limburg, Genk, Belgium; 4 REVAL - Rehabilitation Research Center, Faculty of Rehabilitation Sciences, Hasselt University, Diepenbeek, Belgium; 5 Department of Neurosurgery, University Hospitals Leuven, Leuven, Belgium; 6 Department of Neurosurgery, GasthuisZusters Antwerpen, Wilrijk, Belgium; 7 Department of Neurosurgery, AZ Groeninge, Courtrai, Belgium; 8 Department of Neuropsychology and Psychopharmacology, Faculty of Psychology and Neuroscience, Maastricht University, Maastricht, The Netherlands; 9 School for Mental Health and Neuroscience, Faculty of Health, Medicine and Life Sciences, Maastricht University, Maastricht, The Netherlands; 10 Limburg Brain Injury Centre, Maastricht, The Netherlands; 11 Department of Neurosurgery, Centre Hospitalier de Wallonie picarde, Tournai, Belgium; 12 Department of Neurosurgery, Ziekenhuis Oost-Limburg, Genk, Belgium; 13 Department of Neurosurgery, Noorderhart Mariaziekenhuis, Pelt, Belgium; 14 Department of Orthopedics, AZ Sint-Lucas Gent, Ghent, Belgium; 15 Department of Neurosurgery, Imeldaziekenhuis, Bonheiden, Belgium; 16 Department of Neurosurgery, Regional Hospital Sacred Heart Tienen, Tienen, Belgium; 17 Department of Orthopedics, Noorderhart Mariaziekenhuis, Pelt, Belgium; 18 Experimental Health Psychology, Maastricht University, Maastricht, The Netherlands

**Keywords:** Low back pain, Back surgery, Postoperative pain, Predictors of return to work, Fear of movement-related pain, Avoidance, Fear generalization, Avoidance generalization, Disability, Quality of life

## Abstract

**Background:**

Previous studies indicated that about 20% of the individuals undergoing back surgery are unable to return to work 3 months to 1 year after surgery. The specific factors that predict individual trajectories in postoperative pain, recovery, and work resumption are largely unknown. The aim of this study is to identify modifiable predictors of work resumption after back surgery.

**Methods:**

In this multisite, prospective, longitudinal study, 300 individuals with radicular pain undergoing a lumbar decompression will be followed until 1-year post-surgery. Prior to surgery, participants will perform a computer task to assess fear of movement-related pain, avoidance behavior, and their generalization to novel situations. Before and immediately after surgery, participants will additionally complete questionnaires to assess fear of movement-related pain, avoidance behavior, optimism, expectancies towards recovery and work resumption, and the duration and severity of the pain. Six weeks, 3 months, 6 months, and 12 months after surgery, they will again complete questionnaires to assess sustainable work resumption, pain severity, disability, and quality of life. The primary hypothesis is that (generalization of) fear of movement-related pain and avoidance behavior will negatively affect sustainable work resumption after back surgery. Second, we hypothesize that (generalization of) fear of movement-related pain and avoidance behavior, negative expectancies towards recovery and work resumption, longer pain duration, and more severe pain before the surgery will negatively affect work resumption, pain severity, disability, and quality of life after back surgery. In contrast, optimism and positive expectancies towards recovery and work resumption are expected to predict more favorable work resumption, better quality of life, and lower levels of pain severity and disability after back surgery.

**Discussion:**

With the results of this research, we hope to contribute to the development of strategies for early identification of risk factors and appropriate guidance and interventions before and after back surgery.

*Trial registration* The study was preregistered on ClinicalTrials.gov: NCT04747860 on February 9, 2021.

**Supplementary Information:**

The online version contains supplementary material available at 10.1186/s40359-022-00736-5.

## Background

### Low back pain

Low back pain (LBP) is globally a common health condition and is one of the main reasons people consult a physician [1–4]. It is considered as “*pain, muscle tension or stiffness localized below the costal margin and above the inferior gluteal folds”* [5, p. 1]. Acute LBP is a highly prevalent symptom, affecting 80% of the population at least once in their lifetime [6]. In 10% of the cases, LBP may be caused by a structural pathology such as a fracture, an inflammatory disorder, or a deformity [7]. The most common form of LBP, however, is pain in the lumbar area without a known pathoanatomical problem [8, 9]. For most individuals, the clinical course of LBP is acute and benign [10, 11], with them recovering spontaneously within a few weeks [12]. However, in about one-third of the individuals the pain persists beyond healing time and becomes chronic [11, 13, 14]. Chronic pain is defined as pain that persists or recurs for more than 3–6 months [15, 16]. Due to its persistence, chronic pain can have a detrimental impact on different aspects of life. A subgroup of individuals experiences difficulties in daily life activities and physical impairment [17, 18]. In 2015, the worldwide point prevalence of individuals affected by activity-limiting LBP was estimated at 7.3% [19]. Globally, LBP is also one of the leading causes of years lived with disability [20]. Chronic pain may also affect the psychological wellbeing and mental health of individuals, and often coexists with anxiety and depression [16, 21, 22]. Individuals might, in addition, experience social isolation due to their limitations in maintaining social, family, and sexual relationships [16, 17, 23]. On top of that, chronic pain is a major source of economic burden. Previous studies found that LBP is one of the main reasons for activity limitation, work absence, and even work loss [24–29]. Besides the personal burden, chronic pain entails a huge economic cost for the society due to reduced work efficiency, the use of health care, and sick leave [24, 28, 30–34].

### Back surgery

Over the past decades, the number of back surgeries in Belgium substantially increased. Du Bois et al. [35] reported a rise of 44% from 2001 through 2009 in Belgium. In the past decade, this increase was less pronounced and dropped to 0.44% between 2009 and 2019. From 2017 until 2019, there was even a small decrease of 1.40% [36]. Nevertheless, the number of spine surgeries is still considerable. In 2019, a total of 38,123 spine surgeries were performed. There is however no unequivocal evidence that surgery is the best solution, especially for the treatment of LBP [37]. Studies have found that even after anatomically successful surgery, 10 to 40% of the individuals continue to report pain complaints [38]. A subgroup of individuals undergoing surgery will also be unable to return to work after back surgery. Rates of postoperative return to work differ strongly and vary between 50 to 90% [39]. Recent studies reported that 82% of the individuals were able to return to work 3 months after back surgery [40, 41]. Also in Belgium, Du Bois and Donceel [42] found that 20% of the individuals were unable to resume work 1 year after a lumbar discectomy surgery. Although this considers only a minority of individuals with back pain and radicular pain, they represent a considerable burden to the society [13]. In those individuals, the complaints do not resolve despite anatomical successful surgery. Possible reasons are that psychological and social factors might overrule the effects of surgery, or the specific type of surgery might not have been the right solution for that particular spinal pathology. However, the mechanisms underlying the transition from acute to chronic pain remain poorly understood.

### Mechanisms of chronic pain

The central premise of the current research project is the clinical observation that for some individuals, there seems to be no clear association between recovery of anatomical damage and reduction of pain complaints. The term "Failed Back Surgery Syndrome" (FBSS), for example, refers to persistent axial or peripheral pain following an anatomically successful surgical procedure [43]. In addition, people with chronic back pain often have no identifiable anatomical lesions, or the associated pain severity, affective distress, and disability levels dominate the pain problem [44]. So overall, biological models that seek an explanation in structural and biomedical abnormalities cannot sufficiently explain chronic pain and the functional disability it causes [45–47]. Recovery of complaints should therefore not only be explained by the nature of the injury and the surgical intervention itself, but also by psychological, social, and contextual factors.

### Fear-avoidance model of chronic pain

Over the years, theories considering psychological factors, such as individual beliefs and behavior, have been proposed. An influential theoretical model in this area is the fear-avoidance (FA) model [48–51]. The FA model proposes that chronic pain may develop when pain-related fear and avoidance behavior persist beyond healing time, or when protective responses generalize to novel stimuli (generalization stimuli) resembling the original fear-eliciting stimulus. More specifically, the model proposes that people who interpret pain as a sign of bodily threat, prioritize control of pain that does not go away, leading to fear and avoidance. When negative affect and harm expectations are present, they might increase the engagement in pain control. In the acute pain stage, these protective behaviors serve to reduce or eliminate a justified threat to the body and are considered adaptive. In individuals with chronic pain, however, a pathoanatomical threat (such as injury or spinal pathology) is usually absent, and the costs of avoidance behavior may start to outweigh its benefits. In addition, fear and avoidance are often not restricted to movements/activities that were initially associated with pain but extend to a range of novel (generalization) stimuli. In essence, this *stimulus generalization* is adaptive because it minimizes the necessity to learn everything anew [52], and contains the ability to detect similarities between related stimuli which may contribute to reducing harm in a dynamic environment [53, 54]. Yet, it also bears an increased risk to respond to false alarm threats (“safe stimuli”), which possibly leads to persistent fear and excessive avoidance behavior [54]. Long-term physical inactivity has, in its turn, a negative impact on the musculoskeletal and cardiovascular systems [55–59]. In addition, avoidance may also lead to the withdrawal of positive reinforcers causing interference with valued life activities, which increases negative affect. In its turn, negative affect may worsen the pain even more. In this way, people can get mired in a vicious circle. However, pain-related fear will not always lead to avoidance behavior. The expression of fear of movement-related pain and avoidance behavior is dependent on context. When the value of another life goal outweighs the value of pain control, avoidance behavior can be inhibited [60]. In that case, valued life goals are prioritized and engagement in daily activities will promote recovery [49, 50, 61, 62]. This might be fostered by positive affect and optimism because they enhance goal-directed efforts and stimulate approach instead of avoidance [63].

### Potential predictors

The factors that predict individual differences in outcomes of surgery in terms of postoperative pain, recovery, and resumption of work are still largely unknown. The existing evidence points towards the role of individual beliefs and behavior, sometimes referred to as the “yellow flags” [64, 65]. More specifically, negative emotions [66], catastrophic (mis)interpretations of pain [67], fear of movement-related pain [68, 69], expectancies towards recovery [70], optimism [63, 71] and intolerance of uncertainty [72, 73] appear to significantly influence functioning after back surgery. Linton and Halldén [74] developed a screening tool, called the Örebro Musculoskeletal Pain Questionnaire (ÖMPQ), for the early identification of individuals who risk developing a persistent pain problem. The ÖMPQ was found to be a clinically reliable and valid instrument [75, 76]. In the same vein, Mannion et al. [77], reported that yellow flags significantly contributed to predicting not only the persistency of the pain but also patient outcomes after surgery in general.

### Rationale for the current study

Archer et al. [69] conducted a prospective cohort study in the United States (US) to determine whether preoperative and early postoperative fear of movement-related pain predicts pain, disability, and physical health at 6 months following spinal surgery for degenerative conditions. Their results provided preliminary evidence of the predictive value of early postoperative fear of movement-related pain (six weeks) on the outcome, but not of preoperative fear of movement-related pain [68, 69]. Preoperative fear was found to be a risk factor for increased pain and disability and decreased physical health. However, replications are needed, and the question whether these results generalize to the Belgian health care system is still unanswered.

The aim of this longitudinal, prospective study is to identify modifiable predictors of return to work after back surgery in Belgium. For homogeneity reasons, and because there is often no clear indication for surgery in case of LBP, we chose for the clear-cut indication of radicular pain due to compression. Radicular pain, sometimes as a result of nerve root compression and/or inflammation, is defined as pain that radiates from the back down to the legs and occurs in 3 to 5% of the population, making it one of the most common complaints assessed by spine surgeons [78]. Radicular pain commonly co-occurs with, and might even dominate LBP but can also exist on its own. However, due to their co-occurrence and strong overlap in causes, the two conditions are sometimes difficult to completely disentangle. The primary objective is to investigate the effect of (generalization of) fear of movement-related pain and avoidance behavior on sustainable work resumption after back surgery. The secondary objectives are to investigate the effect of (a) (generalization of) fear of movement-related pain and avoidance behavior on pain severity, disability, and quality of life after back surgery, and (b) expectancies towards recovery and work resumption, duration of the pain before the surgery, severity of the pain before and after the surgery, optimism, and yellow flags for long-term disability and failure to return to work on sustainable work resumption, pain severity, disability, and quality of life after back surgery. The study will be conducted in nine Belgian hospitals.

## Methods

### Design

**Fig. 1:**
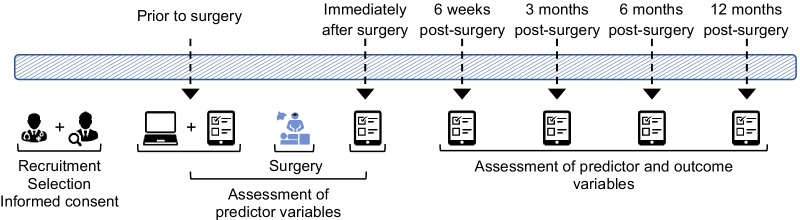
Study design. *Note*. The first icon represents the consult with the surgeon, the second one the consult with the research assistant. The third icon (the computer) represents the assessment of the computer task. The fourth icon (the notepad) represents the assessments of the questionnaire. The fifth and last icon, presented in blue, represents the surgery

This multisite study has a prospective, longitudinal design in which individuals with radicular pain who undergo a lumbar decompression, will be followed prior to surgery up until 1 year post-surgery. Measurements take place prior to surgery (preoperative measurement), immediately after surgery (postoperative measurement), and 6 weeks, 3 months, 6 months, and 12 months post-surgery (follow-up measurements), see Fig. 1. The expected duration of the entire study is approximately 2 years.

### Participants

Three hundred individuals with radicular pain will be recruited. Every participant will be planned for back surgery in one of the nine involved Belgian hospitals (i.e., University Hospitals Leuven, Ziekenhuis Oost-Limburg, Noorderhart Mariaziekenhuis, AZ Groeninge, GasthuisZusters Antwerp, Centre Hospitalier de Wallonie picarde, Imeldaziekenhuis, Regional Hospital Heilig Hart Tienen, and AZ Sint-Lucas Ghent). Inclusion criteria are: (a) age between 18 and 55, (b) proper mastery of the Dutch, English, and/or French language, (c) intermittent or constant invalidating lumbosciatica for less than 1 year prior to the planned surgery, operationalized by being on sick leave on the day of the surgery (documented by the surgeon), (d) radicular pain in the leg(s) due to compression, that will be treated surgically by performing a decompression without it leading to fusion and/or fixation, and (e) self-employed or has an employment contract, and is on sick leave for less than 1 year at the day of the surgery. Reasons for exclusion are (based on self-report): (a) being treated (ambulant or residential) for substance abuse, suicidal ideation, or a psychotic disorder in the year before the consult with the surgeon, (b) at least one back surgery in the last 5 years prior to the planned surgery, and (c) presence of a comorbid condition (e.g. a severe neurological deficit, a pulmonary embolism, permanent paralysis, and another pain problem in the limbs), which may affect the pain, the outcome of the surgery, the duration of the rehabilitation, and/or the participant’s capacity to return to work during the course of the study. At the end of the study, participants will receive a gift voucher worth of maximum 40 euros (at a rate of 10 euros per completed follow-up measurement).

### Sample size

Because there are no previous studies available on the prediction of pain severity and work resumption after back surgery that include all variables in our study, an a priori power analysis for multiple linear regression was conducted. This analysis revealed that, in a model with 12 predictors and a numerator degree of freedom of 14, with a power of 90% (an increase of 0.13 in R^2^), a total sample size of n = 167 is needed to detect a medium effect (effect size f^2^ = 0.1494) at an alpha level of 0.05. Considering potential dropouts from follow-up measurements (conservatively estimated at 50%), the aim for inclusion is set at 300 participants. Power calculations were performed in G*Power version 3.0.10.

## Procedure

### Recruitment and inclusion

Participants will be recruited at the Department of Neurosurgery or Orthopedics of the participating hospitals. The surgeons will screen the participants’ eligibility based on the information that was addressed in the standard anamnestic interview by the neurosurgeon or orthopedic surgeon. When the screening details are not fully known before inclusion, participants’ consent will be requested to ask him/her study-related information to complete the screening.

Before signing the informed consent form, a research assistant will extensively and verbally explain the study protocol. Next, the next study visits will be scheduled, and the participant is requested to indicate in which language (Dutch, English, or French) (s)he wishes to complete the questionnaires and perform the computer task. In addition, participants may also decide to complete the questionnaires of follow-up measurements online or on paper.

### Measurements

For an overview of the measured variables during each assessment, see Table 1.

**Table 1:** Variables measured during each assessment

	Pre-surgery	Immediately after surgery	6 weeks post-surgery	3 months post-surgery	6 months post-surgery	12 months post-surgery
**Outcome variables**						
Self-reported work resumption			✓	✓	✓	✓
Self-reported pain severity	✓	✓	✓	✓	✓	✓
Self-reported disability	✓	✓	✓	✓	✓	✓
Self-reported quality of life	✓	✓	✓	✓	✓	✓
**Predictor variable**						
Self-reported fear of movement-related pain	✓	✓				
Self-reported avoidance behavior	✓	✓				
Generalization of fear of movement-related pain and avoidance behavior (assessed using the computer task)	✓					
Self-reported predictors of long-term disability and failure to return to work (yellow flags)	✓	✓				
Self-reported expectancies towards recovery and work resumption	✓	✓				
**Potential confounding variables**						
Demographic factors - Age - Sex - Relationship status - Highest grade or level of education completed - Duration of incapacity from work	✓					
- Work characteristics (e.g. employment status and rate)	✓		✓	✓	✓	✓
Self-reported work-specific characteristics - Emotional workload - Mental workload - Physical effort - Relationship with supervisor - Relationship with colleagues - Work pleasure - Career opportunities - Organizational commitment - Changing jobs		✓				
Surgery variables (complications during and after surgery)		✓				
Potential influence of COVID-19 - Presence of symptoms - Concerns about health - Physical inactivity	✓					
**Potential moderator variables**						
Self-reported intolerance of uncertainty	✓	✓				
Self-reported optimism	✓	✓				

#### Preoperative measurement

Prior to surgery, the predictor variables, the baseline of the outcome variables, and a selection of potential confounders will be assessed. Therefore, participants will be asked to complete a short computer task and several self-report questionnaires regarding fear of movement-related pain, avoidance behavior, the duration and severity of the pain before the surgery, their expectancies towards recovery and work resumption, optimism, yellow flags for long-term disability and failure to return to work, disability, quality of life, possible influence of the COVID-19 pandemic, and intolerance of uncertainty. We will also administer demographic variables (i.e., age, sex, relationship status, educational attainment, general work characteristics before incapacitated for work, and after work resumption, and start date of incapacity from work). The questionnaires will be implemented in Research Electronic Data Capture (REDCap), a secure web-based software platform [79, 80] and will be completed online on a laptop.

#### Postoperative measurement

In the week after the surgery, the predictor variables, the baseline of the outcome variables after the surgery, and the more specific work characteristics (e.g., workload, relationship with colleagues/supervisor, and pleasure in work) will be assessed. Participants will be asked to complete several self-report questionnaires to assess intolerance of uncertainty, fear of movement-related pain, avoidance behavior, expectancies towards their recovery and resumption of work, optimism, yellow flags for long-term disability and failure to return to work, pain severity, disability, quality of life, and work characteristics. The presence of unexpected complications that occurred during and/or after the surgery will be checked in the medical record of the participants. This measurement will take place during the participant’s recovery time in the hospital. The questionnaires will be implemented in REDCap and will be completed online on a laptop.

### Follow-up measurements: 6 weeks, 3 months, 6 months, and 12 months post-surgery

During the last four measurements, the outcome variables will be assessed. Six weeks, 3 months, 6 months and 12 months post-surgery, the research assistant will contact the participants by e-mail and/or telephone and ask them once again to complete some questionnaires regarding the outcome variables (i.e., sustainable work resumption, pain severity, disability, and quality of life), at home. Participants who experience difficulties completing the questionnaires independently can also complete them with telephone or online support of the research assistant (to prevent drop-out).

## Measurement instruments

### Outcome variables

#### Self-reported work resumption

To measure work resumption, we make a distinction between being fit for work, work resumption, and sustainable work resumption. Being fit for work will be operationalized by not receiving disability benefits. Work resumption will be operationalized by being at work without receiving disability benefits (on a dichotomous scale), and by time to relapse. Time to relapse is defined as the duration of being at work until a participant is on sick leave again. Sustainable work resumption will be operationalized by being at work for at least three consecutive months without receiving disability benefits (on a dichotomous scale). In addition, the duration of work resumption will be measured (on a continuous scale). All these variables will be assessed using custom-made self-report questions. The custom-made questionnaires are presented in Additional file 1. In order to calculate the time to relapse and sustainable work resumption, we will ask the participants to indicate when they were incapacitated from work and when they resumed work. In addition, we will directly ask if the participant resumed work for at least three consecutive months.

#### Self-reported pain severity

Pain severity will be operationalized as the intensity of the pain and will be assessed by one question of the short ÖMPSQ [75]. Participants are requested to answer this item (i.e. *How would you rate the pain that you have had during the past week?*) on a numerical rating scale with labels from 0 = *no pain* to 10 = *pain as bad as it could be*. The score on this item will be used as a measure of pain severity before and immediately after the surgery (as baseline measures) and 6 weeks, 3 months, 6 months, and 12 months post-surgery.

#### Self-reported disability

Disability will be operationalized as the degree to which pain interferes with daily functioning, and will be assessed by the Pain Disability Index (PDI) questionnaire [81]. The PDI is a self-report questionnaire that measures the degree to which pain interferes with functioning in seven areas: i.e., family/home responsibilities, recreation, social activity, occupation, sexual behavior, self-care, and life-support activity [82]. Ratings for each item range from 0 = *no disability* to 5 = *total disability*. A total score will be calculated as a reliable and valid [82] measure of the degree of disability before and immediately after the surgery (as baseline measures) and 6 weeks, 3 months, 6 months, and 12 months post-surgery.

#### Self-reported quality of life

Quality of life will be operationalized as satisfaction with life. It will be measured by the one item of the Riverside Life Satisfaction Scale (RLSS) with the highest factor loading [83]. Participants are requested to answer this item (*I am satisfied with my life overall*) on a 7-point Likert scale ranging from *strongly disagree* to *strongly agree*.

### Predictor variables

#### Self-reported fear of movement-related pain

To measure perceived harmfulness of movements, the following three photographs of the Photograph Series of Daily Activities-short electronic version (PHODA-SeV) [84, 85] were selected: (1) the photograph with the overall highest pain rating (i.e. shoveling soil with a bent back), (2) the photograph with the overall lowest pain rating (i.e. walking through the forest), and (3) the photograph with the highest variance (i.e. trampoline jumping). While a photo is presented on the screen, the participants are asked to rate (on a scale from 0 = *not harmful at all* to 100 = *extremely harmful*) to what extent they feel that the movement on the depicted photo would be harmful to their back if they were to perform it. If there is evidence that the three photographs sufficiently correlate, overall perceived harmfulness will be measured by calculating the average score of the three photographs. Otherwise, the individual score on every photograph will be used as a measure of relative perceived harmfulness.

#### Self-reported avoidance behavior

Avoidance behavior is defined as “*any act or series of actions that enables an individual to avoid or anticipate unpleasant or painful situations, stimuli, or events, including conditioned aversive stimuli”* [86]. In the current study, we will focus on pain-related avoidance behavior, i.e. the tendency to avoid stimuli or activities that could cause pain or pain-related complaints. It will be assessed by the Escape and Avoidance subscale of the short form of the Pain Anxiety Symptoms Scale (PASS-20) [87]. This subscale consists of five items considering thoughts or activities related to avoidance behavior, on which the participants are requested to indicate how often they engage in each of them (on a frequency scale with labels from 0 = *never* to 4 = *always*), and proved to be a valid measure of avoidance behavior [88, 89].

#### Generalization of fear and avoidance behavior

Generalization occurs when novel stimuli (generalization stimuli) evoke responses or behaviors similar to those elicited by a stimulus that naturally evokes a response or behavior (an unconditioned stimulus), or a stimulus that evokes a response or behavior because of its learned association with the unconditioned response (a conditioned response) (Hughes 2011). Such spreading of fear stimuli is following a “better safe than sorry strategy”, which is, in essence, adaptive, but can become maladaptive when there is no threat. Imagine someone experiencing pain when picking up grocery bags. Generalization occurs when the individual starts avoiding putting on pants and mopping the floor, even though these activities were not followed by pain in the past. Generalization, or the spreading of fear, and (generalized) avoidant decision-making to novel movements will be assessed by a recently developed computer task. After approval of the Social and Societal Ethics Committee (SMEC) of KU Leuven (registration number: G- 2018 07 1293) and the Ethical Review Committee Psychology and Neuroscience (ERCPN) of Maastricht University (registration number: Master_189_08_03_2018), this noninvasive computer task was validated in healthy participants at both KU Leuven and Maastricht University. Supporting (cross-site) evidence was found for the construct validity of the paradigm [90]. In this computer task, participants will view digitized images of a person moving in two postures. One posture will be paired with a neutral facial expression and the other posture with a loud human scream and a painful facial expression. Participants will be requested to indicate to what extent they expect the postures to induce pain in the depicted person, and to retrospectively rate their fear towards the postures. Generalization will be tested to intermediate postures (the generalization stimuli) that have a variable similarity between the original two postures, and by assessing pain-expectancy, fear of movement-related pain, and avoidance behavior to the novel stimuli. Pain-expectancy and fear of movement-related pain will be assessed for each stimulus type per block on a continuous rating scale (0–100) ranging from *not at all* to *very much*. Avoidance responses will be calculated as the mean percentage of trials in which participants avoided the painful outcome, for each stimulus type per block.

In contrast to the self-report questionnaires that need introspection from the participant and assess fear and avoidance as a cognitive representation, the computer task assesses fear and avoidance on a behavioral, observable level, is contextualized, and is likely more ecologically valid (being afraid of, and avoid a specific posture because they learned it was aversive). In addition, the computer task also measure generalization (spreading) of fear and avoidance, which is considered a unique predictor of negative outcomes (e.g., [52]). Therefore, we consider both measures complementary.

#### Self-reported predictors of long-term disability and failure to return to work (yellow flags)

Predictors of long-term disability and failure to return to work will be assessed by the short version of the ÖMPSQ [75]. The short version of the ÖMPSQ is a risk assessment tool that consists of 10 items that are scored on a verbal scale, or a numerical rating scale ranging from 0 to 10 (with different labels). This risk assessment tool addresses, among other things, the duration of pain before surgery, pain severity before and after surgery, and expectancies towards recovery and work resumption. The short version of the ÖMPSQ showed to be an accurate and predictive screening tool [75]. A total score will be calculated as a measure of estimated risk for future work disability.

#### Self-reported expectancies towards recovery and work resumption

Participants’ expectancies towards recovery and work resumption will, in addition to the questions of the short form of the ÖMPSQ, be assessed by custom-made self-report questions. We will assess (a) expectancy towards the duration of the pain*,* (b) expectancy towards the severity of the pain, (c) certainty about a decrease in pain, (d) expectancy about the severity of pain after surgery, (e) expectancy about the severity of pain 6 weeks after surgery, (f) expectancy about the severity of pain 3 months post-surgery, (g) expectancy about the severity of pain 6 months post-surgery, (h) expectancy towards resumption of work, (i) expectancy towards the duration until they are able to resume work, (j) certainty about their ability to resume work, (k) expectancy towards sustainability of work resumption. All these items will be rated on a numeric rating scale from 0 to 10 (with different labels). If the participant expects that (s)he will not be able to (sustainably) resume work after surgery, the underlying reason will be interrogated.

### Potential confounding variables

#### Demographic variables

We will register age, sex, relationship status, highest grade or level of education completed, the start date of their incapacity for work, and work characteristics (e.g. type of work, type of contract, and employment rate/hours) before being incapacitated for work and after work resumption. Based on the start date of incapacity for work and the date of work resumption, we will calculate the duration of incapacity for work before surgery. We include this as a potential confounder because a study of Du Bois (2014) showed that 70% of the individuals who were incapacitated for work for longer than 3 months before surgery were unable to resume work 1 year after surgery.

#### Self-reported work-specific characteristics

Work-specific characteristics will be measured by the Experience and Evaluation of Work 2.0 (QEEW2.0) questionnaire [91]. For the purpose of this study, we selected ten subscales: (1) Pace and amount of work, (2) Emotional workload, (3) Mental workload, (4) Physical effort, (5) Relationship with supervisor, (6) Relationship with colleagues, (7) Work pleasure, (8) Career opportunities, (9) Organizational commitment, and (10) Changing jobs (intention to change jobs). These scales will be rated on a 4-point Likert scale ranging from always to never (subscales 1–6), or a 5-point Likert scale ranging from strongly agree to strongly disagree (subscales 7–10). The total score of each subscale will be calculated as a measure of an individual characteristic. The QEEW2.0 is a reliable and valid measurement for research on work, wellbeing, and performance [92].

#### Surgery variables

We will register if complications (i.e. undesirable and unintended events that result from surgery) occur during and/or after the surgery (on a dichotomous scale: yes–no). This data will be monitored by the surgeon and taken from the medical record of the participant.

#### Potential influence of COVID-19

For exploratory reasons, we will examine whether results are influenced by the current COVID-19 pandemic. The participant will be requested to indicate a) to what extend there was concern about health as a result of the COVID-19 pandemic (on a numeric rating scale from 0 = *not at all* to 10 = *very much*), b) to what extent physical activity was limited due to the COVID-19 pandemic (on a numeric rating scale from 0 = *I have not at all been less physically active* to 10 = *I have very much been less physically active*), and c) if two or more of the typical COVID-19 symptoms were experienced simultaneously in the past 6 months (on a dichotomous scale: yes–no).

### Potential moderator variables

#### Self-reported intolerance of uncertainty

Intolerance of uncertainty (IU) is defined as “*the tendency of an individual to consider the possibility of a negative event occurring unacceptable, irrespective of the probability of occurrence*” [91, p. 105], which will be measured by the 12-item Intolerance of Uncertainty Scale (IUS-12) [93]. Participants are requested to indicate to what extent the statements apply to them (on a 5-point Likert scale ranging from 1 = *not at all characteristic of me* to 5 = *entirely characteristic of me)*. A total score will be calculated as a measure of responses to uncertainty. The IUS-12 has shown to be a reliable and valid questionnaire with satisfactory psychometric properties [93–96] in both clinical as non-clinical study samples [97]. IU is included as a potential moderator of the relationships between the predictors and outcome variables. In previous research, IU was found to be a potential risk factor for persistent pain, and excessive and inflexible avoidance, which both have been found to be associated with mental disorders [72, 98]. In addition, IU was found to be related to higher levels of fear and non-adaptive cognitions (e.g., worrying, ruminating, and catastrophizing) in uncertain situations, causing people who are intolerant of uncertainty to be more likely to interpret ambiguous information in a threatening way [72, 93, 98, 99]. Therefore, we assume intolerance of uncertainty to be a risk or vulnerability factor in the relation between the predictors and outcome variables of interest.

#### Self-reported optimism

Optimism is defined as “*the attitude that good things will happen and that people's wishes or aims will ultimately be fulfilled*” (American Psychological Association, n.d.). Trait optimism will be measured by the Life Orientation Test-Revised (LOT-R) questionnaire [100]. The LOT-R consists of 10 statements, on which participants are requested to indicate to what extent they agree (on a 5-point Likert scale ranging from *strongly disagree* to *strongly agree),* and has shown to have satisfactory psychometric properties [101]. A total score will be calculated as a measure of generalized optimism. Also optimism will be considered a potential moderator of the relationships between the predictors and outcome variables. Previous research has shown that optimism is related to improved psychological and physical well-being [102, 103]. It has shown that optimists typically experience less pain, better adjust to pain because of adaptive behavior, and have a better quality of life [71, 102, 104]. In addition, empirical evidence has shown that optimism is related to better outcomes after major invasive and minor elective surgeries [71]. Therefore, we assume optimism to have a protective function in the relation between the predictors and outcome variables of interest.

## Statistical analysis plan

Following an intent-to-treat approach, data from all included participants will be analyzed. Descriptive statistics will be used to explore the mean and median scores, and standard deviations of all measures. Depending on the type of dependent variable, we will apply Cox proportional-hazards regression analysis with time-dependent covariates or multiple (logistic) regression analysis, starting with a univariate analysis, followed by a multivariate analysis. If statistical power allows, interaction effect between time and all the predictor variables will also be tested to evaluate if the effect of the predictor variables is consistent in time. Additionally, we will check for site effects with the mixed model regression approach. All variables will be examined for the assumptions required for parametric analyses. Results will be evaluated at *p* < 0.05 significance level. Effect sizes (i.e., odds ratios) and post hoc power will be calculated when appropriate.

### Primary objectives

Logistic regressions will be performed to examine the effect of preoperative as well as postoperative fear of movement-related pain and avoidance behavior (and their generalization), expectancies towards recovery, expectancies towards work resumption, presence of yellow flags, on return to work at 6 weeks, 3 months, 6 months, and 12 months post-surgery. Next, a time-dependent Cox regression will be performed to examine the effect of the predictor variables on return to work over time. The same analyses will be conducted for the sustainability of work resumption at 3 months, 6 months, and 12 months post-surgery as a dependent variable.

Multiple linear regression analyses will be carried out to examine the effect of the predictor variables on the duration to resume work over time. Log-rank test and Kaplan–Meier plots will be applied to compare rates of work resumption between participants with different profiles (i.e. at work or not during the different measurement moments).

All interaction effects, including the predictor*time interactions, will be explored. Following potential confounders will be included as covariates in each analysis: age, sex, work characteristics, duration of incapacity for work, surgery complication, and the potential influence of the COVID-19 pandemic.

### Secondary objectives

Multiple linear regressions will be carried out to examine the effect of (generalization of) fear of movement-related pain and avoidance behavior, expectancies towards recovery and work resumption, and ÖMPSQ score on pain severity, disability, and quality of life. Interaction effects, including the interaction between the predictors and time, will be explored. Following potential confounders will be included as covariates in each analysis: age, sex, work characteristics, duration of incapacity for work, surgery complications, and the potential influence of the COVID-19 pandemic.

### Moderator effects

To check if optimism and intolerance of uncertainty moderate the relationship between the predictors and the outcome variables, we will include these in interaction terms in the relation between the predictor variables and the outcome variables.

## Discussion

Low back pain and radicular pain are highly prevalent health problems in western societies [14]. In most individuals, the pain is benign and will recover spontaneously over time. However, in about one-third of the individuals, the pain persists beyond healing time and becomes chronic, being responsible for a considerable burden on both the individual and the society. Over the past decades, the number of back surgeries substantially rose in several Western countries [35, 105]. This increase became less pronounced in Belgium and evolved in a more stable way with even a small decrease in the past years. However, there seems to be a global threat of overuse of healthcare, including the execution of unnecessary surgeries [106]. This is concerning because the execution of surgeries does not only entail a considerable cost for the society and a waste of healthcare resources, it also exposes individuals to potential hazardous consequences and long-lasting rehabilitation [45, 106–108]. Previous studies, for example, reported that 20% of the individuals are unable to return to work after back surgery. Studies in the US also found that individuals who underwent surgery continued to have poorer physical and mental functioning compared with the general US population [109, 110]. In addition, a systematic review showed that recurrent back pain may occur in 15–25% of individuals 2 years after undergoing a discectomy for lumbar disc herniation [111]. As mentioned before, the relatively high number of non-desirable outcomes might be partially caused by poor diagnostic evaluation or misclassification of diagnoses or treatments (e.g. selection of patients without a clear indication for that specific treatment), leading to unnecessary and inadequate surgery [112–115]. In most individuals with LBP, there is actually no causal relationship between underlying pathology and pain severity [14, 45]. There is also no unequivocal evidence that surgery is the best solution for treatment of LBP (in the long term) [37]. Based upon these findings, the Belgian guidelines regarding the management of LBP and radicular syndrome were recently revised. These guidelines are moving away from pharmacological and surgical treatments, and place greater emphasis on self-management and non-invasive interventions such as exercise and psychological treatments [116, 117]. In addition, a risk stratification, with attention to yellow flags, to predict the risk for chronic pain is usually added as an important step in the treatment of LBP [117, 118].

Nevertheless, postoperative outcomes are often variable, differ among individuals and interventions, and depend on different factors (both personal, social, and contextual). Based upon the finding that biological models cannot sufficiently explain postoperative outcomes, many studies investigated potential risk and beneficial factors [29, 66, 119]. However, the specific factors that predict individual trajectories in postoperative outcomes, recovery, and resumption of work are largely unknown. The available evidence points towards the role of dysfunctional beliefs and avoidance behaviors. Therefore, we aim to identify psychological predictors that promote or impede (functional) recovery and sustainable work resumption after back surgery. In order to keep the participant sample as homogeneous as possible, we chose to include individuals with radicular pain due to compression for which surgery can be indicated [78].

So far, the empirical evidence on the influence of personal factors on work resumption is strongly limited because it is almost exclusively based on cross-sectional studies. To our knowledge, the proposed study is novel for its longitudinal, prospective design, and the use of a computer task measuring behavioral responses in addition to self-reports. A longitudinal prospective design enables us to investigate if and when the predictors affect post-surgical work resumption, pain severity, disability, and quality of life.

Practical issues of this study comprise a higher risk of dropout over time, which might impede high levels of data completeness. In addition, the multicentric design might make it more difficult to warrant consistent execution of the study protocol across all participating sites. Besides that, most predictors are assessed based on self-report measures, which are momentary and susceptible to several biases and socially desirable answers. To compensate for these biases, the computer task was developed for the assessment of generalization of pain-related fear and avoidance behavior.

With the results of this research, we hope to gain more insight into the factors affecting recovery and work resumption after back surgery and contribute to the development of strategies for early identification of risk factors, and appropriate guidance and interventions before and after back surgery. In its turn, these interventions may lead to better recovery and adjusted and sustainable reintegration into the labor market.

## Supplementary Information

**Additional file 1.** . Custom-made questionnaires. Questionnaires that were developed for this study (pdf).

## Data Availability

After the study is completed, the datasets used and/or analyzed during the current study will be available from the corresponding author on reasonable request.

## Abbreviations

ERCPN: Ethical Review Committee Psychology and Neuroscience
FA: Fear-avoidance
FBSS: Failed Back Surgery Syndrome
IUS: Intolerance of Uncertainty
LBP: Low back pain
LOT-R: Life Orientation Test-Revised
ÖMPSQ: Örebro Musculoskeletal Pain Screening Questionnaire
PASS: Pain Anxiety Symptoms Scale
PDI: Pain Disability Index
PHODA-SeV: Photograph Series of Daily Activities-short electronic version
QEEW: Questionnaire on the Experience and Evaluation of Work
REDCap: Research Electronic Data Capture
RLSS: Riverside Life Satisfaction Scale
SMEC: Societal Ethics Committee
TRACE: Centre For Translational Psychological Research
VAS: Visual Analogue Scale
WABS: Work After Back Surgery
