# Supplementary material for: Generalization of fear of movement-related pain and avoidance behavior as predictors of work resumption after back surgery: a study protocol for a prospective study (WABS)
Source: BMC Psychol. 2022 Feb 22;10:39. doi: 10.1186/s40359-022-00736-5 (PMC8862001; doi:10.1186/s40359-022-00736-5)
Supplement: Supplementary file 1 — Additional file 1. . Custom-made questionnaires. Questionnaires that were developed for this study (pdf). [file 40359_2022_736_MOESM1_ESM.pdf]

Custom-made questionnaires

1. PRE- AND POSTOPERATIVE MEASUREMENTS

A. Expectancy towards pain intensity

The first questions are about the pain for which you will undergo surgery.

1. To what extent do you **expect** that the pain will decrease as a result of the surgery?

0 = I do NOT AT ALL expect that the pain will decrease — 10 = I VERY MUCH expect that the pain will decrease

0	1	2	3	4	5	6	7	8	9	10
○	○	○	○	○	○	○	○	○	○	○

2. How **sure** are you that the pain will decrease as a result of the surgery?

0 = I am NOT AT ALL sure that the pain will decrease — 10 = I am VERY sure that the pain will decrease

0	1	2	3	4	5	6	7	8	9	10
○	○	○	○	○	○	○	○	○	○	○

0 = No pain — 10 = Pain as bad as it could be

	0	1	2	3	4	5	6	7	8	9	10
3. How much pain do you expect to have in **the week** after the surgery?	○	○	○	○	○	○	○	○	○	○	○
4. How much pain do you expect to have **6 weeks** after the surgery?	○	○	○	○	○	○	○	○	○	○	○
5. How much pain do you expect to have **3 months** after the surgery?	○	○	○	○	○	○	○	○	○	○	○
6. How much pain do you expect to have **6 months** after the surgery?	○	○	○	○	○	○	○	○	○	○	○
7. How much pain do you expect to have **12 months** after the surgery?	○	○	○	○	○	○	○	○	○	○	○

B. COVID-related items

The following questions are related to COVID-19.

1. To what extent have you been concerned about **your health** as a result of the COVID-19 pandemic?

0 = Not at all | 10 = Very much

0	1	2	3	4	5	6	7	8	9	10
○	○	○	○	○	○	○	○	○	○	○

2. To what extent have you been less physically active due to the COVID-19 pandemic?

0 = I have NOT AT ALL been less physically active | 10 = I have been VERY MUCH less physically active

0	1	2	3	4	5	6	7	8	9	10
○	○	○	○	○	○	○	○	○	○	○

3. Did you experience two or more of the typical COVID-19 symptoms (such as fever, dry cough, fatigue, shortness of breath, sore throat, chest pain, body aches, and a changed sense of taste or smell) simultaneously in **the past 6 months**?

○ Yes
○ No

C. Work-related items

The last questions are about your work.

1. Tick all the boxes that apply to you.

☐ I am an employee (= employed by a company) → **Go to question 2**
☐ I am a civil servant (= employed by the government) → **Ga to question 3**
☐ I am self-employed (= no formal employment contract) → **Go to question 4**

2. What type of employment contract do you have as an employee?

○ Laborer (blue-collar/physical work)
○ Clerk (white-collar work)

3. What is your employment rate?

☐ **% as employee**

☐ **% as civil servant**

If you are not self-employed → go to question 6

---

4. Self-employed:

○ In main occupation

○ In secondary occupation

---

5. How many hours per week do you work on average as a self-employed worker?

☐ hours

---

6. Are you currently working **without** also receiving disability benefits (= benefits because you are unable to work) for this period?

○ Yes, I am working and I am not receiving disability benefits for this period
→ Go to question 7

○ No, I am working and I am also receiving disability benefits for this period
→ Go to question 7

○ No, I am not working and I am not receiving disability benefits for this period
→ Go to question 7

○ No, I am not working and I am receiving disability benefits for this period
→ Go to question 8

---

7. From which date will you stop working **and** will you receive disability benefits for that period because you are unable to work? (dd/mm/yyyy)

……… / ……… / ………………

If you receive disability benefits → go to question 9
If you do not receive disability benefits → go to question 10

---

8. Since when did you stop working **and** are you receiving disability benefits for that period because you are unable to work? (dd/mm/yyyy)

……… / ……… / ………………

---

9. Why do you receive disability benefits?

Because I cannot work due to …

- ○ … the pain/complaints related to the back surgery I will undergo
- ○ … another reason

---

10. To what extent do you **expect** to officially return to work in the year following the surgery, without also receiving disability benefits for that period because you are (partially) unable to work?

0 = I do NOT AT ALL expect to return to work — 10 = I VERY MUCH expect to return to work

0	1	2	3	4	5	6	7	8	9	10
○	○	○	○	○	○	○	○	○	○	○

---

11. How **sure** are you that you will return to work in the year following the surgery, without also receiving disability benefits for that period?

0 = I am NOT AT ALL sure that I will return to work — 10 = I am VERY sure that I will return to work

0	1	2	3	4	5	6	7	8	9	10
○	○	○	○	○	○	○	○	○	○	○

If you answered <u>question 71</u> with a number from 0 to 4 → go to question 12
If you answered <u>question 71</u> with a number from 5 to 10 → go to question 13

---

12. Why do you think that you will not return to work in the year following the surgery **without** also receiving disability benefits for that period?

- ○ Because of the pain/complaints related to the back surgery I will undergo
- ○ Because of another reason

→ Go to question 18

---

13. From which date do you expect to officially return to work **without** also receiving disability benefits for that period? (dd/mm/yyyy)
If you intend to resume/start multiple professions, please fill out the date of the profession you intend to resume/start <u>first</u>.

……… / ……… / ………………

14. Why do you think that you will not return to work **before** that date?

- ○ Because of the pain/complaints related to the back surgery I will undergo
- ○ Because of another reason

---

15. To what extent do you **expect** to work for **at least 3 consecutive months** (= without being on sick) after resuming work, without also receiving disability benefits for that period because you are (partially) unable to work?

0 = I do NOT AT ALL expect to work for 3 consecutive months

10 = I VERY MUCH expect to work for 3 consecutive months

0	1	2	3	4	5	6	7	8	9	10
○	○	○	○	○	○	○	○	○	○	○

---

16. How **sure** are you that you will work for **at least 3 consecutive months** (= without being on sick leave) after resuming work, without also receiving disability benefits for that period because you are (partially) unable to work?

0 = I am NOT AT ALL sure that I will work for 3 consecutive months

10 = I am VERY sure that I will work for 3 consecutive months

0	1	2	3	4	5	6	7	8	9	10
○	○	○	○	○	○	○	○	○	○	○

If you answered question 15 with a number from 0 to 4 → go to question 17
If you answered question 15 with a number from 5 to 10 → go to question 18

---

17. Why do you think that you will not work for 3 consecutive months (= without being on sick leave) without also receiving disability benefits for that period because you are (partially) unable to work?

- ○ Because of the pain/complaints related to the back surgery I will undergo
- ○ Because of another reason

---

18. Do you have any comments about the questionnaire? Please write them down below.

2. FOLLOW-UP MEASUREMENTS AFTER 6 WEEKS

A. Work resumption

The first questions are about your work.

1. Do you receive disability benefits because you are currently (partially) unable to work?

- ○ Yes → **Go to question 3**
- ○ No → **Go to question 2**

2. Since when do you no longer receive disability benefits? (dd/mm/yyyy)
   Please fill out the first day of the period for which you no longer receive disability benefits.

……… / ……… / ………………

→ **Go to question 4**

3. Why do you receive disability benefits?

Because I am unable to work due to …

- ○ … the pain/complaints related to the back surgery at the beginning of this study
- ○ … the pain/complaints related to another (later) back surgery
- ○ … another reason

4. Did you officially return to work*?
   *partially or fully

- ○ Yes → **If you do not receive disability benefits: go to question 6**
  → **If you as well receive disability benefits: go to question 7**
- ○ No → **Go to question 5**

5. Because of what are you not working?

- ○ Because of the pain/complaints related to the back surgery at the beginning of this study
- ○ Because of the pain/complaints related to another (later) back surgery
- ○ Because of another reason

→ **Go to question 11**

6. Since when are you working **without** also receiving disability benefits for that period? (dd/mm/yyyy)
   If you resumed/started multiple professions, please fill out the first date.

……… / ……… / ………………

7. Which of the following professions did you resume or start?
Multiple answers are possible.

- ○ Profession as an employee → **Go to question 8**
- ○ Profession as a civil servant → **Go to question 8**
- ○ Self-employed profession → **Go to question 9**

---

8. What is your employment rate?

[ ] **% as employee**

[ ] **% as civil servant**

If you are not self-employed → go to question 10

---

9. How many hours per week do you work on average as a self-employed worker?

[ ] hours

---

10. Do you practice the same profession as before the surgery? If you practiced multiple professions within the same category (employee, civil servant or self-employed worker) before the surgery, please indicate whether you still practice the same **main profession** as before the surgery.

	Yes	No	Not applicable
**As employee**	○	○	○
**As civil servant**	○	○	○
**As self-employed worker**	○	○	○

---

3. FOLLOW-UP MEASUREMENTS AFTER 3 MONTHS

3.1 Participant was incapacitated at the time of the previous measurement

A. Work resumption

The first questions are about your work.

1. Do you receive disability benefits because you are currently (partially) unable to work?

○ Yes → **Go to question 3**
○ No → **Go to question 2**

2. Since when do you no longer receive disability benefits? (dd/mm/yyyy)
Please fill out the first day of the period for which you no longer receive disability benefits.

.... / .... / ....

→ **Go to question 6**

3. Why do you receive disability benefits?

Because I am unable to work due to …

○ … the pain/complaints related to the back surgery at the beginning of this study
○ … the pain/complaints related to another (later) back surgery
○ … another reason

4. Did you receive disability benefits for the entire period **from the last measurement moment** (approximately a month and a half ago) **until today**?

○ Yes, I received disability benefits for the entire period → **Go to question 6**
○ No, I also did not receive disability benefits for a period in between → **Go to question 5**

5. For which period(s) did you no longer receive disability benefits?
Please fill out as many periods as applicable.

**Period 1**	**Period 2**	**Period 3**	**Period 4**
From (dd/mm/yyyy)	From (dd/mm/yyyy)	From (dd/mm/yyyy)	From (dd/mm/yyyy)
……./……./………	……./……./………	……./……./………	……./……./………
Until (dd/mm/yyyy)	Until (dd/mm/yyyy)	Until (dd/mm/yyyy)	Until (dd/mm/yyyy)
……./……./………	……./……./………	……./……./………	……./……./………

6. Did you officially return to work*?
   *partially or fully

- ◯ Yes ⟶ **If you <u>don't</u> receive disability benefits: go to question 8**
  ⟶ **If you also receive disability benefits: go to question 9**
- ◯ No ⟶ **Go to question 7**

---

7. Because of what are you not working?

- ◯ Because of the pain/complaints related to the back surgery at the beginning of this study
- ◯ Because of the pain/complaints related to another (later) back surgery
- ◯ Because of another reason

⟶ **Go to question 13**

---

8. Since when are you working **without** also receiving disability benefits for that period? (dd/mm/yyyy)
   If you resumed/started multiple professions, please fill out the <u>first</u> date.

……… / ……… / ………………

---

9. Which of the following professions did you resume or start?
   Multiple answers are possible.

- ◯ Profession as an employee ⟶ **Go to question 10**
- ◯ Profession as a civil servant ⟶ **Go to question 10**
- ◯ Self-employed profession ⟶ **Go to question 11**

---

10. What is your employment rate?

☐ **% as employee**

☐ **% as civil servant**

If you are not self-employed ⟶ go to question 12

---

11. How many hours per week do you work on average as a self-employed worker?

☐ hours

---

12. Do you practice the same profession as before the surgery? If you practiced multiple professions within the same category (employee, civil servant or self-employed worker) before the surgery, please indicate whether you still practice the same **main profession** as before the surgery.

	Yes	No	Not applicable
**As employee**	○	○	○
**As civil servant**	○	○	○
**As self-employed worker**	○	○	○

3.2 Participant was <u>not incapacitated</u> at the time of the previous measurement

A. Work resumption

The first questions are about your work.

1. Do you receive disability benefits because you are currently (partially) unable to work?

○ Yes → **Go to question 2**
○ No → **Go to question 4**

2. Why do you receive disability benefits?

Because I am unable to work due to …

○ … the pain/complaints related to the back surgery at the beginning of this study
○ … the pain/complaints related to another (later) back surgery
○ … another reason

3. Since when do you receive disability benefits again? (dd/mm/yyyy)
Please fill out the first day of the period for which you receive disability benefits again.

……… / ……… / ………………

→ **Go to question 7**

4. Did you no longer receive disability benefits for the entire period **from the previous measurement moment** (approximately a month and a half ago) **until today**?

○ Yes, I did not receive disability benefits for the entire period → **Go to question 7**
○ No, I also received disability benefits for a period in between → **Go to question 5**

5. For which period(s) did you for receive disability benefits again?
   Please fill out as many periods as applicable.

**Period 1**	**Period 2**	**Period 3**	**Period 4**
From (dd/mm/yyyy)	From (dd/mm/yyyy)	From (dd/mm/yyyy)	From (dd/mm/yyyy)
……./……./………	……./……./………	……./……./………	……./……./………
Until (dd/mm/yyyy)	Until (dd/mm/yyyy)	Until (dd/mm/yyyy)	Until (dd/mm/yyyy)
……./……./………	……./……./………	……./……./………	……./……./………

6. Why did you receive disability benefits for this/these period(s)?
   Please fill out as many periods as applicable.

Period 1: Because I was unable to work due to …

- ○ … the pain/complaints related to the back surgery at the beginning of this study
- ○ … the pain/complaints related to another (later) back surgery
- ○ … another reason

Period 2: Because I was unable to work due to …

- ○ … the pain/complaints related to the back surgery at the beginning of this study
- ○ … the pain/complaints related to another (later) back surgery
- ○ … another reason

Period 3: Because I was unable to work due to …

- ○ … the pain/complaints related to the back surgery at the beginning of this study
- ○ … the pain/complaints related to another (later) back surgery
- ○ … another reason

Period 4: Because I was unable to work due to …

- ○ … the pain/complaints related to the back surgery at the beginning of this study
- ○ … the pain/complaints related to another (later) back surgery
- ○ … another reason

7. Did you officially return to work*?
   *partially or fully

- ○ Yes → **If you do not receive disability benefits: go to question 9**
  → **If you also receive disability benefits: go to question 10**
- ○ No → **Go to question 8**

8. Because of what are you not working?

- ○ Because of the pain/complaints related to the back surgery at the beginning of this study
- ○ Because of the pain/complaints related to another (later) back surgery
- ○ Because of another reason

→ Go to question 14

---

9. Since when are you working **without** also receiving disability benefits for that period? (dd/mm/yyyy) If you resumed/started multiple professions, please fill out the first date.

……… / ……… / ………………

---

10. Which of the following professions did you resume or start? Multiple answers are possible.

- ○ Profession as an employee **→ Go to question 11**
- ○ Profession as a civil servant **→ Go to question 11**
- ○ Self-employed profession **→ Go to question 12**

---

11. What is your employment rate?

[ ] **% as employee**

[ ] **% as civil servant**

If you are not self-employed → go to question 13

---

12. How many hours per week do you work on average as a self-employed worker?

[ ] hours

---

13. Do you practice the same profession as before the surgery? If you practiced multiple professions within the same category (employee, civil servant or self-employed worker) before the surgery, please indicate whether you still practice the same **main profession** as before the surgery.

	Yes	No	Not applicable
**As employee**	○	○	○
**As civil servant**	○	○	○
**As self-employed worker**	○	○	○

---

4. FOLLOW-UP MEASUREMENTS AFTER 6 AND 12 MONTHS

4.1 Participant was incapacitated at the time of the previous measurement

A. Work resumption

→ All questions are conform follow-up measurements after 3 months.

4.1 Participant was incapacitated at the time of the previous measurement

A. Work resumption

→ All questions are conform follow-up measurements after 3 months.
